# The initiation of mitochondrial DNA replication

**DOI:** 10.1042/BST20230952

**Published:** 2024-06-17

**Authors:** Yi Liu, Haibin Liu, Fan Zhang, Hong Xu

**Affiliations:** 1 Hubei Jiangxia Laboratory, Wuhan 430200, China; 2 National Heart, Lung and Blood Institute, NIH, Bethesda, MD 20892, U.S.A.

**Keywords:** exonucleases, mitochondria, mtDNA, PPR, replication, RNA polymerase

## Abstract

Mitochondrial DNA replication is initiated by the transcription of mitochondrial RNA polymerase (mtRNAP), as mitochondria lack a dedicated primase. However, the mechanism determining the switch between continuous transcription and premature termination to generate RNA primers for mitochondrial DNA (mtDNA) replication remains unclear. The pentatricopeptide repeat domain of mtRNAP exhibits exoribonuclease activity, which is required for the initiation of mtDNA replication in *Drosophila*. In this review, we explain how this exonuclease activity contributes to primer synthesis in strand-coupled mtDNA replication, and discuss how its regulation might co-ordinate mtDNA replication and transcription in both *Drosophila* and mammals.

## Introduction

**Figure 1.**
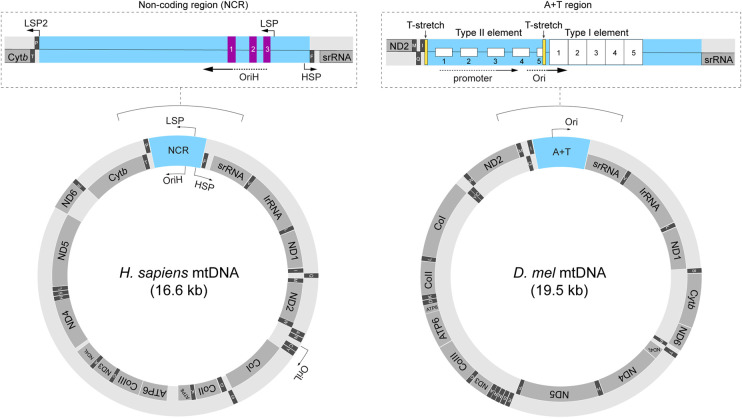
Organization of the human (left) and *D. melanogaster* (right) mitochondrial genomes. Schematic of human mtDNA showing all genes, non-coding region (NCR), promoters of both heavy and light strands (HSP, LSP, and LSP2), and replication origins of both heavy and light strands (OriH, OriL). Dashed box showing an enlarged version of the human mtDNA NCR to emphasize locations of LSP, conserved sequence blocks (CSB 1–3), and OriH. Schematic of *D. melanogaster* mtDNA showing all genes, the non-coding A/T rich region (A + T region), and replication origin (Ori). Dashed box showing an enlarged version of A/T rich region to illustrate the locations of two conserved repetitive sequence elements (Type I and Type II elements), promoter region, Ori, and two sequences of tandem thymidine (T-stretch). Please note that the exact locations of promoter sequence and replication origin have not been precisely mapped in *Drosophila*. Arrows of origins and promoters denote the directions of replication or transcription, respectively.

A typical animal cell contains hundreds to thousands of copies of small, closed circular DNA (mitochondrial DNA, mtDNA) inside the mitochondrial matrix [1]. Animal mtDNA is extremely compact and lacks introns or regulatory sequences, with the exception of a non-coding region (NCR) that contains *cis*-elements necessary for mtDNA replication and transcription [2,3] (Figure 1). Human mtDNA encodes 13 core components of the oxidative phosphorylation system, as well as RNAs, including 2 rRNAs and 22 tRNAs, that are necessary for the translation of these protein-coding genes within the organelle [4,5]. Mitochondria operate under dual genetic control [6]. Aside from the 13 proteins encoded by mtDNA, all remaining mitochondrial proteins, including those required for mtDNA maintenance and gene expression, are encoded by the nuclear genome [2,6].

The core mtDNA replisome comprises of mtDNA helicase (Twinkle) [7,8], mitochondrial single-strand DNA-binding protein (mtSSB) [9], and the heterotrimeric replicase Polγ [10,11], consisting of a single catalytic subunit, Polγ-α, and two accessory subunits, Polγ-β [12,13]. The obvious omission here is a primase that can synthesize short RNA oligonucleotides to prime DNA replication, as DNA synthesis cannot be initiated *de novo* [14]. However, no primase has been identified in mitochondrial proteomes [15,16]. It is now widely accepted that mtDNA replication is coupled to the transcription carried out by the mitochondrial RNA polymerase (mtRNAP) [17–19]. Transcriptional regulations on mtDNA are best characterized in mammals [20]. Mitochondrial genes on both strands, designated as the heavy (H) and light (L) strands due to their distinct buoyant densities [21], are transcribed by human mtRNAP (POLRMT) as polycistronic RNAs, which are subsequently processed into individual mRNAs, rRNAs, and tRNAs [12]. Promoters for the light strand (LSP) and the heavy strand (HSP) that account for the majority of mitochondrial transcripts have been mapped into NCR [21] (Figure 1). A second light strand promoter (LSP2) with substantially weaker activity than that of LSP has also been identified [22,23], although its physiological significance remains unknown. mtRNAP is a single-subunit RNA polymerase, structurally similar to bacteriophage T7 RNA polymerase [24,25]. Different from T7 RNA polymerase, mtRNAPs have a long N-terminal domain that contains two pentatricopeptide repeat (PPR) motifs [25], degenerate 35-amino-acid sequences of tandem repeats [26]. All PPR proteins are located in either mitochondria or chloroplasts and involved in organellar RNA processing and editing [27], while the biochemical basis of these actions is not well understood.

**Figure 2.**
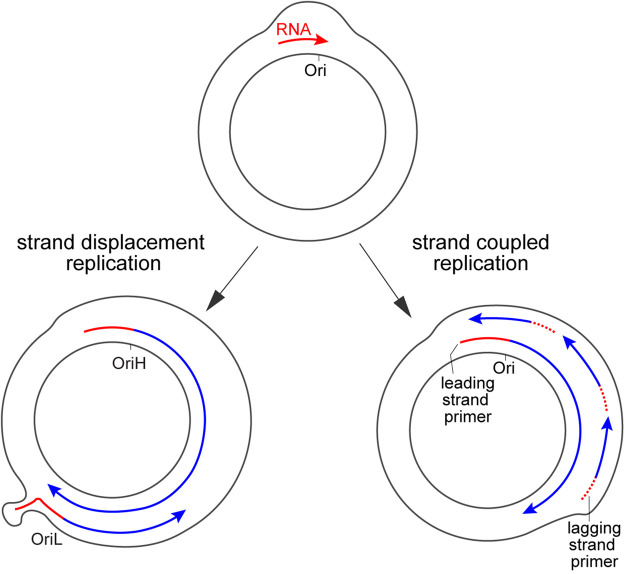
Schematics of asymmetric (strand displacement, left) and strand-coupled (right) models of mtDNA replication. Both models begin with the transcription by mtRNAP, generating RNA species (red line) that will be prematurely terminated near the replication origin and further processed for priming mtDNA replication. According to the strand displacement model in mammals, the replication of the heavy strand is initiated at the OriH. Until the fork passing the light strand origin, the exposed parental H strand forms a stem–loop structure near OriL, which can be recognized by POLRMT to initiate the transcription, resulting in short RNA oligos (red line) to prime the replication of the light strand in the opposite direction. In the strand-coupled replication, the replication fork moves unidirectionally. The lagging strand is replicated discontinuously and requires short RNA oligos (dashed red lines) to prime the synthesis of Okazaki fragments.

Currently, various models of mtDNA replication have been proposed in different cell types and organisms [28]. Mammalian mtDNA is mainly replicated via the strand displacement model, in which H and L strands are replicated asynchronously [29]. mtDNA replication begins at H strand origin (OriH) in NCR and proceeds unidirectionally for about two-thirds of the circular genome, exposing the origin of L strand (OriL), which allows the replication of L strand in the opposite direction [6] (Figure 2). Another prevailing model of mtDNA replication is the strand-coupled replication, also known as the θ model that has been demonstrated in various species including *Drosophila* [28,30]. In this model, the mtDNA replication is initiated in NCR and two strands are replicated concurrently (Figure 2). While the leading strand is replicated continuously, the lagging strand DNA synthesis is initiated at multiple sites, producing series of Okazaki-fragment-like intermediates [30]. In both models, mtDNA replication is preceded by the transcription in NCR carried out by mtRNAP, which generates RNA molecules that are necessary to initiate mtDNA replication [17–19] (Figure 2).

Additional mechanisms and detailed processes of mtDNA replication have been extensively discussed elsewhere [20,28,31]. In this review, we provide a brief overview of protein factors, sequence elements, and molecular processes necessary for the initiation of mtDNA replication in the two aforementioned models of replication: strand displacement replication and strand-coupled replication, based on studies in mammals and fruit flies, respectively. We highlight the essential roles of mtRNAP's exoribonuclease activity in generating RNA primers and its potential contribution to coordinating mtDNA replication and transcription.

## Strand-coupled mtDNA replication in *Drosophila*

**Figure 3.**
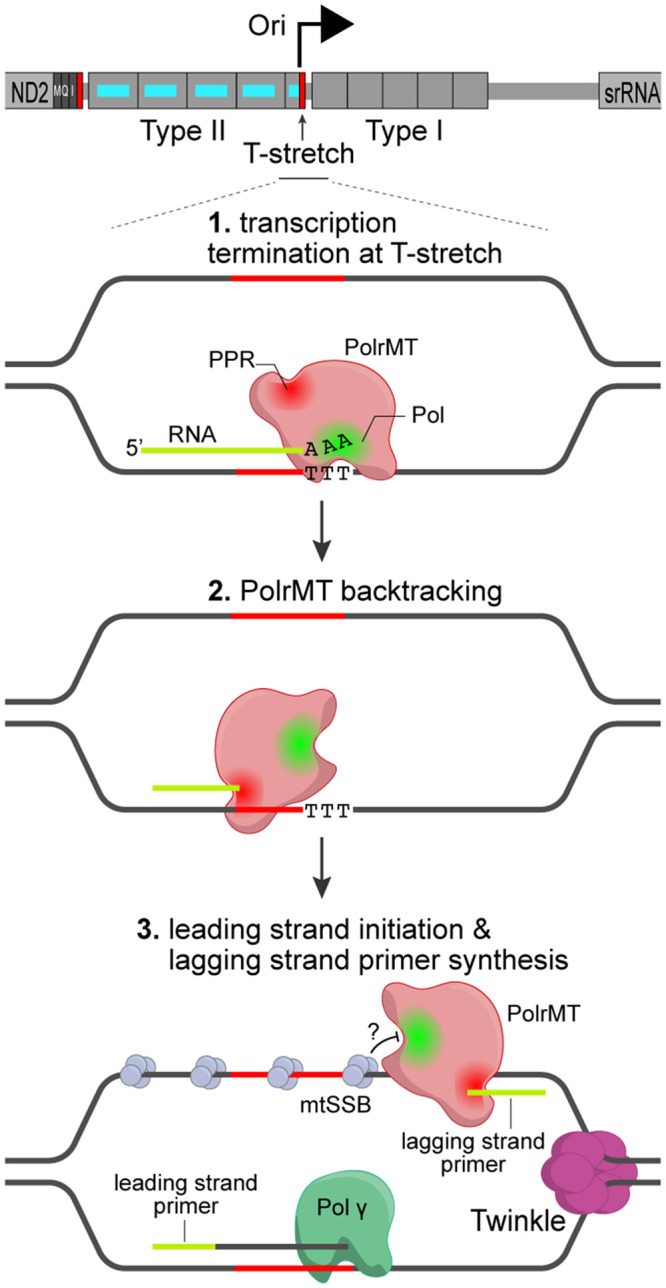
Proposed model for the initiation of mtDNA replication in *D. melanogaster*. The replication origin (Ori) has been roughly mapped to the center of the A–T region, near a stretch of 21 thymine nucleotides (T-stretch, red block) that is flanked by two types of repeated elements. A 300-bp highly conserved sequence element (cerulean bars) in Type II repeats potentially contains a promoter for PolrMT. We propose sequential processes that lead to the initiation of synthesis of both the leading and the lagging strands in *Drosophila*. (1) The transcription is terminated at the T-stretch near Ori. (2) Due to the intrinsic instability of A-U pairs, the 3′ end of RNA could dissociate from the DNA template, which triggers PolrMT backtracking to degrade the nascent RNA strand. (3) Short RNA oligos would be generated to prime the leading strand replication by Polγ. In the progressing fork, PolrMT would start transcription on the exposed ssDNA that is coated with mtSSB. When the elongating PolrMT encounters mtSSB, which inhibits the polymerase activity, the PPR domain would degrade the nascent transcripts, generating short RNA oligos to prime the lagging strand synthesis.

*Drosophila* mtDNA encodes the same set of genes as its human counterpart. The overall gene organization and structure of these two genomes are quite similar (Figure 1), except that the NCR of *Drosophila* mtDNA is slightly longer than in human. An early study using transmission electron microscopy (TEM) analysis on replicating mtDNA molecules suggested strand-asynchronous mtDNA replication in *Drosophila* [32]. However, a recent study using 2D gel electrophoresis analysis on purified replication intermediates demonstrated that *Drosophila* mtDNA is predominantly replicated via strand-coupled model, with a minor population of asynchronized replication [30]. The replication origin has been roughly mapped to the center of the NCR [30,32], often referred to as the ‘A/T rich region' due to its extremely high adenine (A) and thymine (T) contents [33] (Figure 1), and the replication fork moves unidirectionally toward the *srRNA* gene [30]. The *Drosophila* mtDNA is transcribed to four primary long polycistronic transcripts [34], and one transcript covers almost half of the A/T rich region and extends to rRNA genes [35]. Hence, a promoter must be located near the center of A/T rich region as well (Figure 1). Comparative sequence analysis on mitochondrial genomes of several *Drosophila* species identified a 300-bp highly conserved sequence element, and a stretch of thymine nucleotides (T-stretch) located near the presumptive replication origin [34,36]. Transcription by RNA polymerase is prone to termination at an A/T rich region [37]. Notably, a similar T-stretch at the replication origin of bacterial colicin E1 plasmid is the signal for terminating transcription by the RNA polymerase, which allows the transition from primer synthesis to the initiation of DNA replication [38]. Thus, the transcription by mtRNAP could be prematurely terminated at the T-stretch, resulting in RNA oligos to prime the leading strand synthesis by Polγ (Figure 3). Consistent with this proposition, *Drosophila* mtRNAP (PolrMT) is indispensable for the *de novo* mtDNA synthesis [17], highlighting a conserved role of mtRNAP in initiating mtDNA replication across phylogeny [17–19]. However, the molecular mechanism underlying the switch from mitochondrial transcription to mtDNA replication is not fully understood. Additionally, it is puzzling how PolrMT, which transcribes mtDNA to long polycistronic transcripts, can generate series of short RNA oligos to prime the lagging strand synthesis in the strand-coupled replication.

## How does PolrMT's exoribonuclease activity contribute to the initiation of mtDNA replication?

The polymerase domain of mtRNAP is highly homologous to T7 RNA polymerase [24,25]. While T7 RNA polymerase produces the full length, run-off transcripts on linear DNA templates *in vitro*, mtRNAPs mainly produce short RNA oligos [17,18]. The PPR domain of *Drosophila melanogaster* PolrMT possesses a 3′–5′ exoribonuclease activity, which enables the generation of short oligonucleotides to prime DNA replication *in vitro* [17]. The PPR domain of animal mtRNAPs contains two PPR motifs, each of which consists of two α-helices, forming a helix–turn–helix fold [26]. While the enzymatic basis of PPR domain degrading RNA remains to be determined, this characteristic structure of the helix–turn–helix fold appears to be necessary. A single amino-acid-residue substitution of Glu423 to Pro in one PPR motif of PolrMT completely abolishes the exonuclease activity while leaving the polymerase activity unaffected [17]. Importantly, this PolrMT variant largely restores mitochondrial transcription but not *de novo* mtDNA replication, demonstrating that PolrMT's exonuclease activity is necessary for initiating mtDNA replication [17].

In DNA/RNA hybrids, the configuration of the RNA 3′ end impacts the kinetics of degradation by PolrMT, as an unpaired RNA 3′ end is degraded much faster than a paired end [17]. The intrinsic instability of A-U pairs makes the elongating RNA strand more likely to dissociate from the template DNA when PolrMT reaches the T-stretch. This would generate an unpaired 3′ end on nascent RNA, favoring its degradation by the PPR domain. Once the degradation is initiated, PolrMT is highly processive on DNA/RNA hybrids and rapidly degrades the RNA strand to short oligonucleotides, ranging in length from 5 to 10 nucleotides [17]. This could be the underlying mechanism of PolrMT generating RNA primers for the leading strand synthesis in *Drosophila* (Figure 3). Notably, overexpression of the exonuclease-deficient PolrMT variant in adult fruit flies markedly increases incorporation errors in mitochondrial transcripts [17], indicating a contribution of PolrMT's exonuclease activity in maintaining transcription fidelity.

As a core component of mtDNA replisome, mtSSB stabilizes the single-stranded DNA (ssDNA) in replication bubbles and stimulates the processivity of Polγ [9,18,39]. Human mtSSB has an inhibitory effect on POLRMT's transcription in an *in vitro* assay [18]. In the strand-coupled replication in *Drosophila*, when an elongating PolrMT encounters mtSSBs on the lagging strand in a progressing replication fork, mtSSB may also inhibit PolrMT's polymerase activity and allow the PPR domain to degrade the newly synthesized RNA, generating primers for lagging strand synthesis (Figure 3).

## Unsettled issues regarding the initiation of the strand displacement mtDNA replication in mammals

Mitochondrial replication has been extensively studied in mammals [12,20,31]. The strand displacement mode of mtDNA replication is preceded by the transcription of the L strand at LSP, carried out by mtRNAP, POLRMT [20]. The binding of mammalian mitochondrial transcription factor, TFAM to LSP bends DNA and recruits POLRMT onto DNA through direct protein–protein interaction [40]. Subsequently, mitochondrial transcription factor B2, TFB2M binds to this protein–DNA complex, unwinding DNA double helix and enabling POLRMT to initiate transcription [41–43]. The mitochondrial transcription elongation factor (TEFM) stabilizes elongating POLRMT on the template DNA and stimulates its polymerase activity to transcribe nearly the full length of the genome [44,45]. There is a stretch of Guanine-rich sequence in a conserved sequence block (CSB II), ∼100 bp downstream of LSP in NCR [46]. It has been proposed that Guanines on both the nascent RNA strand and the non-template DNA in CSB II could form a hybrid DNA–RNA G-quadruplex [47,48], potentially slowing down POLRMT and causing premature termination in the absence of TEFM [48,49]. An isoform of RNase H1, an endoribonuclease that cleaves RNA strand in RNA–DNA hybrids, is localized to mitochondria [50]. Loss of RNase H1 leads to mtDNA depletion, and it has been proposed that RNase H1 can digest prematurely terminated transcripts, generating short RNA oligos to prime H strand replication [51]. A fraction of transcription from LSP is terminated at the CSB I region, generating a non-coding 7S RNA that can block the loading of POLRMT onto mtDNA [52]. Hence, 7S RNA may provide a negative feedback regulation on both mitochondrial transcription and mtDNA replication in mammals. The origin of the L strand has been mapped to a region ∼11 kb downstream of the LSP, located between two tRNA genes, *mt-tRNA^Cys^* and *mt-tRNA^Asn^* [53,54]. After the replication fork passes OriL, the exposed parental H strand is predicted to form a stem–loop structure [53,55], which can be recognized by POLRMT to initiate transcription [56,57]. The transcription by POLRMT on ssDNA templates spanning OriL is predominantly terminated near a stretch of tandem adenine nucleotides *in vitro*, generating short RNA oligos that could potentially prime L strand synthesis in the opposite direction [57,58].

It should be noted that the aforementioned model of H strand initiation does not fully align with the genetic studies in animal models [51,59,60]. Ablation of *RNase H1* leads to mtDNA replication stalling but not a lack of initiation in mice [51]. Similarly, knockdown of *RNase H1* in *Drosophila* does not impair the initiation of mtDNA replication; instead, it results in an accumulation of replication intermediates [59]. Additionally, a variant of RNase H1 with enhanced enzyme activity is defective in primer formation [51], contradicting its proposed role in processing prematurely terminated transcripts to prime mtDNA replication. Furthermore, *de novo* mtDNA replication is reduced, not increased in *Tefm* knockout mice [60]. Hence, the mechanism of H strand initiation remains unsettled. Additionally, the proposed model of initiation at OriL is purely based on *in vitro* studies. The PPR domain of human POLRMT also exhibits a 3′–5′ exoribonuclease activity [17]. In an *in vitro* assay, the transcription by a POLRMT variant lacking the PPR domain is not prematurely terminated at CSB II [17], suggesting a potential role of POLRMT's exoribonuclease activity in mediating the switch from mitochondrial transcription to mtDNA replication.

## Future directions

PolrMT possesses two distinct activities, polymerase activity and 3′–5′ exoribonuclease activity that are carried out by its polymerase domain and PPR domain, respectively. These two domains potentially compete for the same substrate, the 3′ end of the nascent RNA strand, as the full-length PolrMT has weaker exonuclease activity than the PPR domain alone [17]. Hence, the coordination between PolrMT's polymerase and exonuclease activities likely determines the fate of newly synthesized RNAs, whether they are continuously elongated to polycistronic RNAs or partially degraded for priming mtDNA replication. Sequence elements on mtDNA, metabolites, or protein factors that interact with PolrMT and modulate either of these two activities would likely be involved in determining the switch between mitochondrial transcription and mtDNA replication. In mammals, mtSSB inhibits the transcription by POLRMT and promotes the initiation of mtDNA replication [18,39]. It would be interesting to test whether mtSSB might also regulate PolrMT's exonuclease activity or polymerase activity in *Drosophila*. Additionally, exploring potential regulations on mtRNAPs by mtSSB could shed light on the initiation of mtDNA replication in both mammals and *Drosophila*. We postulate that PolrMT's preference for an unpaired RNA 3′ end may lead to the premature termination of transcription at the T-stretch. This idea could be readily tested in an *in vitro* transcription assay, which may also determine the promoter sequence in the A/T rich region.

Mitochondrial transcription is essential for oxidative phosphorylation, and hence the switch between mtDNA replication and transcription regulates cellular energy metabolism [61]. ATP can stimulate the initiation of transcription by mtRNAP [62,63], leading to a proposal that ATP levels and mitochondrial transcription may form a feed-forward loop to maintain cellular energy output [64]. However, it is rather counterintuitive that a highly energized cell needs to further enhance energy metabolism. In bacteria, the initiator protein, DnaA binds to ATP and the accumulation of ATP-DnaA molecules leads to the initiation of DNA replication [65]. This mechanism allows bacteria transiting from cell growth to cell division depending on cellular energy status. Given the endosymbiotic origin of mitochondria, ATP levels might also signal mtDNA replication that is loosely coupled to the nuclear genome replication in mitotic cells [66,67]. ATP-stimulated mitochondrial transcription may mainly be used for initiating mtDNA replication. It will be intriguing to test whether NTPs, dNTPs, and other metabolic intermediates could influence either mtRNAP's polymerase activity or its exonuclease activity. These studies may unveil how mtDNA replication and transcription are regulated by cellular energy metabolism and physiological status.

## Perspectives

- Mitochondrial DNA maintenance and gene expression are paramount for cellular energy metabolism. Elucidating the molecular mechanisms initiating mtDNA replication is not only important for a better understanding of mitochondrial biogenesis, but may also help to develop potential therapies for mtDNA depletion syndrome or related metabolic diseases.
- Due to a lack of dedicated primase in mitochondria, mtDNA replication is coupled to mitochondrial transcription. In mammals, transcription by POLRMT can be prematurely terminated near both OriH and OriL, generating RNA species to initiate the replication of the H strand and L strand, respectively, in the model of strand displacement replication. The exact mechanisms of premature terminations at both origins are not fully understood. Human POLRMT exhibits exoribonuclease activity that contributes to the premature termination near the OriH and likely regulate the switch from continuous transcription to mtDNA replication. In *Drosophila*, the exoribonuclease activity is indispensable for PolrMT to generate short RNA oligos necessary for priming the lagging strand synthesis. The inherent instability of A-U pairs and PolrMT's preference on unpaired 3′ end of RNA strands on DNA/RNA hybrids could also contribute to the premature termination of PolrMT near the T-stretch, leading to the generation of RNA molecules that initiate the leading strand replication in the strand-coupled model.
- The mechanisms underlying the switch between continuous transcription and primer formation remain to be defined in both mammals and *Drosophila*. The future discovery of protein factors, sequence elements, and potentially metabolites that can influence mtRNAP's polymerase or nuclease activities will lead to a better understanding of mtDNA replication initiation and its coordination with mitochondrial translation. This line of research may also unveil how mitochondrial biogenesis adapts to cellular energy demand and physiological status.

## Abbreviations

HSP: promoters for the heavy strand
LSP: promoters for the light strand
LSP2: second light strand promoter
mtRNAP: mitochondrial RNA polymerase
mtSSB: mitochondrial single-strand DNA-binding protein
NCR: non-coding region
PPR: pentatricopeptide repeat
ssDNA: single-stranded DNA
TEFM: transcription elongation factor
